# Agile Requirements Engineering and Software Planning for a Digital Health Platform to Engage the Effects of Isolation Caused by Social Distancing: Case Study

**DOI:** 10.2196/19297

**Published:** 2020-05-06

**Authors:** Edward Meinert, Madison Milne-Ives, Svitlana Surodina, Ching Lam

**Affiliations:** 1 Digitally Enabled PrevenTative Health Research Group Department of Paediatrics University of Oxford Oxford United Kingdom; 2 Department of Primary Care and Public Health Imperial College London London United Kingdom; 3 Skein Ltd London United Kingdom; 4 Institute of Biomedical Engineering University of Oxford Oxford United Kingdom

**Keywords:** telemedicine, information science, data science, COVID-19, coronavirus, public reporting of healthcare data, health care quality, access and evaluation, aged, mental health, exercise, cellphone, artificial intelligence, agile, requirements engineering, social distancing, digital health, app

## Abstract

**Background:**

Social distancing and shielding measures have been put in place to reduce social interaction and slow the transmission of the coronavirus disease (COVID-19). For older people, self-isolation presents particular challenges for mental health and social relationships. As time progresses, continued social distancing could have a compounding impact on these concerns.

**Objective:**

This project aims to provide a tool for older people and their families and peers to improve their well-being and health during and after regulated social distancing. First, we will evaluate the tool’s feasibility, acceptability, and usability to encourage positive nutrition, enhance physical activity, and enable virtual interaction while social distancing. Second, we will be implementing the app to provide an online community to assist families and peer groups in maintaining contact with older people using goal setting. Anonymized data from the app will be aggregated with other real-world data sources to develop a machine learning algorithm to improve the identification of patients with COVID-19 and track for real time use by health systems.

**Methods:**

Development of this project is occurring at the time of publication, and therefore, a case study design was selected to provide a systematic means of capturing software engineering in progress. The app development framework for software design was based on agile methods. The evaluation of the app’s feasibility, acceptability and usability shall be conducted using Public Health England's guidance on evaluating digital health products, Bandura’s model of health promotion, the Reach Effectiveness Adoption Implementation Maintenance (RE-AIM) framework and the Nonadoption, Abandonment and Challenges to the Scale-up, Spread and Suitability (NASSS) framework.

**Results:**

Making use of a pre-existing software framework for health behavior change, a proof of concept was developed, and a multistage app development and deployment for the solution was created. Grant submissions to fund the project and study execution have been sought at the time of publication, and prediscovery iteration of the solution has begun. Ethical approval for a feasibility study design is being sought.

**Conclusions:**

This case study lays the foundations for future app development to combat mental and societal issues arising from social distancing measures. The app will be tested and evaluated in future studies to allow continuous improvement of the app. This novel contribution will provide an evidence-based exemplar for future app development in the space of social isolation and loneliness.

## Introduction

### Background

Social distancing measures have been put in place to reduce social interaction and slow transmission of a recently discovered novel coronavirus disease (COVID-19) [1]. For older people (defined as adults 65 years or older), self-isolation presents particular challenges for physical activity, mental health, and social relationships [2]; continued regulated social distancing could have a compounding impact on these concerns. In this population, physical activity is associated with a greater than 22% reduction in mortality [3]. The implications of social distancing could have unintended adverse mental and physical health outcomes by advancing social isolation, loneliness, and sedentary lifestyles [4].

Preliminary research suggests that the implementation of “lockdown” measures significantly reduces the doubling rate of COVID-19 [5]. As such, governments throughout Europe have implemented various degrees of containment measures to enact social distancing and limit exposure of populations to casual contact to slow the spread of the disease [6]. In Western democracies such as the United Kingdom, governments are finding it challenging to ensure people stay at home during the lockdown and do not exploit the daily exercise and essential shopping rules, especially as the weather starts to improve [6]. Such violations reduce the effectiveness of lockdown measures. There is no practical way of monitoring the status of people with minor symptoms, especially those who are unable to get tested. These patients are not considered in the national statistics and cause the overall patient numbers to be underestimated, making evidence-based policy making unreliable. These circumstances prevent health systems from having accurate information on inbound case numbers, which makes managing case volume challenging and often results in resource overloads.

Preliminary data suggests that the rate of patients requiring hospitalization due to COVID-19 increases dramatically with age. Based on data from COVID-19 cases in China, approximately 16% of people aged 60 years or older who become infected are expected to need hospital care [7]. Case fatality rates for people 60 years or older are estimated (from international cases) to be 4.5%, compared to 1.4% for those younger than 60 years [8]. In 2015, 32.1% of the European population was aged 65 and older [9]. Although public health strategies to slow the spread of the disease may prove useful, they also introduce a burden of social isolation at home and in the hospital. Even before COVID-19, older people—particularly those who spend the majority of their time alone—were at a higher risk of social isolation [10,11]. Social isolation—having few social interactions—has been linked to an increased risk for a range of physical and mental health problems, including cardiovascular disease, stroke, dementia, and depression [11]. Loneliness, the subjective perception of inadequate social connection, has also been independently associated with depressive symptoms [2,10,12]. The current limitations on social contact exacerbate the vulnerability of older people to social isolation, loneliness, and the associated mental and physical effects on well-being.

Current solutions include schemes to have volunteers call and talk with older people or use established social media platforms as mechanisms to promote social interaction [13]. The challenge with each of these approaches is, although they may be useful in establishing baseline contact, they are generic approaches that are not customized to the nature of the current problem. Additionally, systems often have a limited theoretical basis in evidence-based health behavior change frameworks. Many families are attempting to use social media platforms (eg, Skype, Facebook Messenger, or Zoom) to connect with older members of their families [14]. However, these platforms were not designed to address the needs of older users in both application and user experience, and have limited functions. Prevention of social isolation can be achieved through one-on-one communication, group interactions, and structural interventions; although the sustainability of intervention is a crucial factor in long-term effectiveness [11].

This pandemic creates an overwhelming demand on hospitals that is challenging to coordinate proactively [15]. Managing the inbound flow of care delivery resources is difficult because health systems (primary, secondary, and tertiary care) are not linked in a capacity to draw on data sources that could indicate trends on potential new cases [16]. Design standards that would promote interoperability necessary to integrate patient-facing and hospital-wise care systems are still emerging [17]. However, the need for such integration is more urgent than ever because the ability to plan for care based on health system capacity is especially vital during this time.

### Aim and Objectives

The purpose of this paper is to provide details to the user needs and subsequent system design for the Activating Digital to Support Social Distancing COVID-19 Aware Family Engagement (ADAPT-CAFÉ) solution. As this is an in-progress initiative, it is hoped that peer review and dissemination of the project design and associated implementation details will generate further discussion and reflection on digital health solutions being used as tools for engagement during and after the pandemic.

### Project ADAPT-CAFÉ

This project team will design, develop, and deploy a digital health mobile app to provide a means of assisting families and peer groups in maintaining contact with older people. The app will use goal setting and online communities to encourage positive nutrition, physical activity, and virtual interaction during social distancing. Although there is mixed evidence on the effectiveness of electronic interventions for loneliness in older people [18], through an examination of strengths and weaknesses of previous studies, we hypothesize that it is possible to build a successful intervention with a user-centered and behavioral change theory-based design. Anonymized data from the app will be aggregated with other real-world data sources to develop a machine learning algorithm, with the objective of improving identification of patients with COVID-19 and tracking the real time use by health systems.

We aim for the immediate impact to be 27,450 users (approximately .01% of the older population in the United Kingdom, France, and Sweden) within the United Kingdom and the European Union. The target impact will be achieved over a series of months following the app’s release, implemented via targeted paid advertising (eg, search engines, social media, news, television, and radio) and placement on app stores to promote uptake. The project impact will be quantitatively measured by registration and app use data. The solution platform could also be exploited worldwide if the initial implementation achieves its objectives.

### Solution Overview

The solution is a combination of:

- A mobile digital health app that provides older people, their families, and peers with a structured medium for social interaction.
- An analytics reporting engine that provides anonymized data from the app on potential cases, which can be used to anticipate hospital demand.

**Figure 1:**
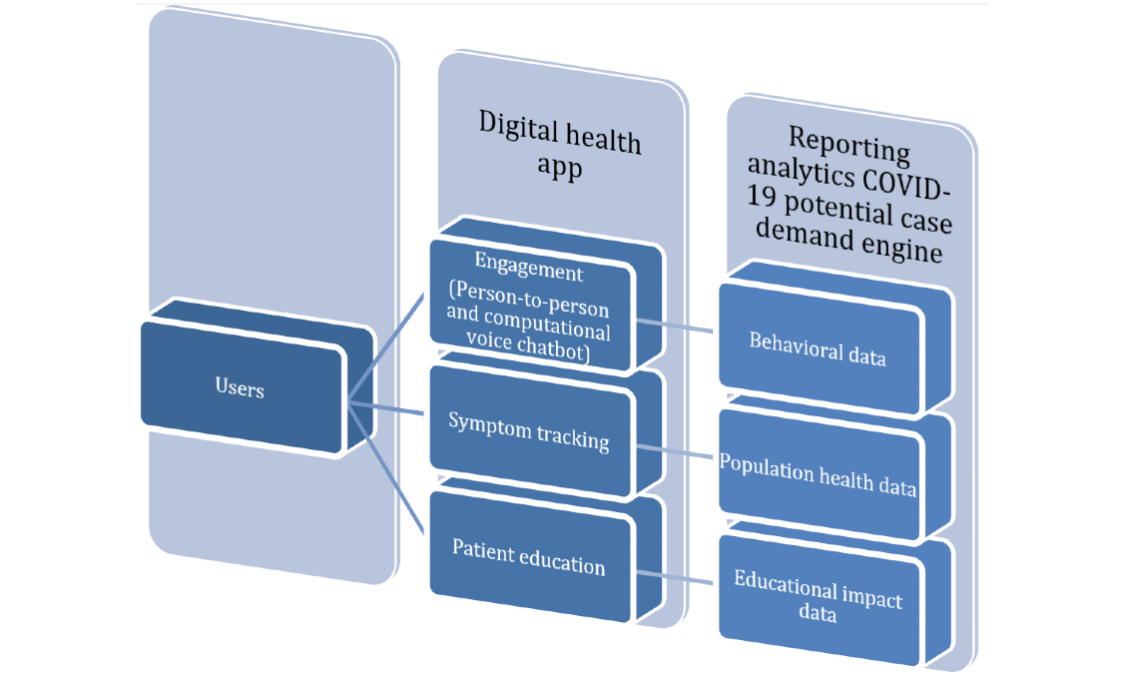
ADAPT-CAFÉ high-level implementation features. ADAPT-CAFÉ: Activating Digital to Support Social Distancing COVID-19 Aware Family Engagement; COVID-19: coronavirus disease.

Both technologies are based on pre-existing software frameworks that will be modified for this use case, allowing for rapid app development and deployment for immediate impact. Figure 1 shows the ADAPT CAFÉ high-level implementation features.

### Design Extensibility

A vital aspect of this system is to provide the capability of enabling user engagement across multiple priorities. Although the proposed use case is focused on older users, hospitalized patients of all ages are increasingly socially isolated [19]. It is also a significant public health concern that many patients with cardiac conditions (particularly those with heart attacks) are presenting too late or not at all during the pandemic as a result of shielding. The solution is designed to be extensible to these and other implementation scenarios to encourage engagement and promote positive behaviors during social isolation and health system interaction.

### Solution Overview: Mobile Digital Health App

The digital health app targets older users and their family members. Previous research suggests that there is mixed evidence that information communication technology interventions can be useful in reducing social isolation in older people [10]. The critical challenge in these technologies is mitigating issues centered on app usability and accessibility. This project will implement a simple-to-use interface with built-in accessibility functions such as font size adjustment, a chatbot (text and voice), a voice control assistant, and voice messaging with family members.

The app will allow family members to remotely access the interface of the older user’s app so that family members can teach the user how to use it without face-to-face contact. Suggested daily checkup messages can be sent from family members and friends in-app or via text messaging depending on the older users’ preferences. Social interaction suggestions for virtual family gatherings, physical activity, and healthy nutrition will be provided with cues in-app to encourage consistency and sustainable uptake. Gamification, achievement, and in-app rewards will also be incorporated to incentivize users to schedule and plan for activities with family members.

Daily automated voice messages to collect well-being data from users will also be used to actively track real world data, including activities, location, and symptom data, in the user population. Natural language recognition will be used to analyze responses from users to collect relevant epidemiological and geographical data and allow earlier identification and treatment of infected older people. This will prevent symptoms from worsening and thus reduce death rates. The data generated by the app, including voice and text interactions, geographical location, and other activity patterns, will be anonymized, securely stored, and analyzed for insights to be extended as a signal used by health systems as predictors of potential new patients with COVID-19.

There are over 238 million older citizens throughout Europe [9]. Although the initial implementation will be provided in English, French, and Spanish, this app has the potential to be extended to older populations around the world.

The app will be developed using the behavior change wheel (BCW) framework [20]. This theory was designed to guide the development of behavior change interventions by outlining three behavioral components—capability, opportunity, and motivation—that interact to affect behavior. Effective interventions can be developed by evaluating which of these components need to be changed and how to achieve a target behavior [20]. The BCW framework also links these behavioral components with various intervention strategies (eg, persuasion, education) so that intervention types can be chosen concerning the behavioral components to be changed [20]. This framework will be used as a basis for evaluating what is preventing individuals from engaging in digital social interactions, physical activity, and symptom reporting so that the interventions incorporated in the mobile app will target those aspects specifically. The behavior change techniques (BCT) taxonomy will also be used to provide a clear description of the specific techniques being used in the app [21]. The clarity that this taxonomy provides will enable easy and rapid evaluation and adaptation of the BCTs used in the app in response to interim data so that the app will use the most effective BCTs in this context.

### Solution Overview: Reporting Analytics COVID-19 Potential Case Demand Engine

Anonymized data from the ADAPT CAFÉ app will be a source of real-world evidence for understanding people’s movement within lockdown regions as well as the occurrence of symptoms within the user group. This data can be aggregated with other data sources to provide a complete picture of the geographical region and insights into both the social and physical needs of users under lockdown. This data will be used as a source of information to create dynamic stratified patient demand forecasts with both machine learning transferred parameters and an agent-based simulation of patient demographics. This data will develop a geographical model superimposed on a health system’s capacity to serve care, which will be analyzed against current and future availability of care delivery resources. These features will enable the capability to quickly see critical paths and provide the health system with the ability to plan and allocate providers and assets. This will enable the ability to simulate “what-if” scenarios and enable operational decision support for logistical placement, resource assignment, and management.

GE Healthcare's Command Center (GE Healthcare) provides a “wall of analytics” that draws on data from multiple systems within a clinical care setting; this data is displayed across a hospital and is accessible via tablets and mobile devices [22]. Advanced algorithms are used to help staff predict and resolve bottlenecks in care delivery before they occur, recommending actions to enable faster, more responsive patient care and better allocation of resources [22]. GE is using a significance and inferiority ranking approach [23] to develop an agent-based simulation to stratify people and networks of interactions. This insight could enable the transfer of learnings from one region to another to tailor demand capacity testing. This aim would be to identify trends by gaining an understanding of behaviors that are leading to infections and associated precursors. This will enable hospital information flow to be bidirectional: the creation of a propensity for future demand by demographic and an increase to the effectiveness of the digital health ADAPT-CAFÉ app at delaying or preventing infections that health systems do not have the capacity to serve due to overwhelming demand.

Anonymized real world evidence gathered through ADAPT-CAFÉ will be used by GE’s Command Centre technology or as a stand-alone dashboard and data source for integration with other clinical care data systems to improve the general model predictive power and optimize clinical resources for the treatment of patients with COVID-19.

## Methods

### Case Study Design

In-depth data on the effectiveness and acceptability of the proposed app will be collected using a case study method [24]. Focused qualitative and quantitative research on the user's experience of this app will allow further development to target-specific issues identified. The case study development will follow seven stages, outlined in Table 1.

**Table 1:** Case study framework (based on [24,25]).

Number	Stage	Corresponding agile stage	Outcomes
1	Plan	Discovery phase	Description of problem, user journeys, and data aggregation approach
2	Design	Alpha phase	Construction of research design and linkage of research questions, data, and criteria for evaluation and synthesis Development and design of app prototype based on the behavior change wheel theoretical framework [20], which will be iteratively updated based on new incoming evidence from steps 4 and 5
3	Prepare	Alpha phase	Drafting, approval, and execution of study ethics followed by the performance of user recruitment protocols Design of short-term and long-term app evaluation protocols
4	Collect	Beta phase	Deployment of a beta version of the app and conduction of qualitative semistructured interviews and in-app surveys, and collection of app use data.
5	Analyze	Beta phase	Iterative user data analysis for continuous app improvement
6	Create	Live phase	Finalize app for publishing on Google and Apple app stores
7	Share	Live phase	Ongoing paid advertising to promote uptake of the app Creation of interim and long-term reports based on iteratively collected data and a final report of the impact of the app on reducing social isolation, improving well-being, and providing useful tracking data Publishing of the reports in a peer-reviewed journal.

### Nonadoption, Abandonment, Scale-Up, Spread, and Sustainability Framework

Comprehensive planning for the potential sustained impact and long-term use of this platform will be done using the nonadoption, abandonment, scale-up, spread, and sustainability (NASSS) framework [26]. Adaptation of the solution use case will commence with consideration of the issues concerning social isolation and older users, with user-centered design and patient and public engagement in solution design to ensure the appropriateness of the solution design. Considerations of the broader health system, organizational, value proposition, and longer-term adaptation over time shall be planned and iterated throughout the app life cycle using the 7 stages for the framework and recorded in subsequently published reports on the implementation of the system.

### Agile Software Development Process

**Figure 2:**
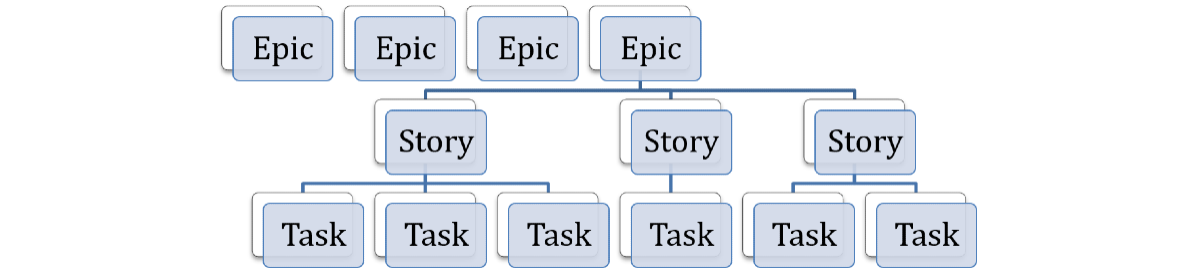
Sample of agile software requirements planning.

The software product development is following the agile framework defined by the UK Government Service Manual [27], as well as lean methodology [28] and iterative design and development sprints. The approach relies on reuse and iterative improvements of the code and user experience elements, in particular via deploying open-source tools and codebase, conducting fast validation with real users, and maintaining consistent performance measurements against predefined key performance indicators, including adoption and retention metrics. Figure 2 shows a sample of agile software requirements planning.

The discovery phase aims to efficiently define requirements via a structured process, with subsequent iterative build and testing during the alpha, beta, and live phases. Aligned with the Service Manual agile framework guidelines [29], the accessibility standards and principles are integrated into the system design and development process. This approach also allows the resulting insights and technology to be replicable, scalable, and transferable to extend the solution to the adjacent problem areas. 

### Evaluation: Study Design

A feasibility study will be initiated and last 12 months; this will include a 1-month evaluation and intervention refinement (ending by June 27, 2020), and an 11-month implementation and follow-up (commencing June 28, 2020, and concluding June 1, 2021). During the implementation period, the research team will prepare for subsequent larger-scale studies should interim results indicate study feasibility, adoption, and usability of the app (by December 31, 2020). The study is centered on the app and excludes the reporting engine; the basis for this is that successful uptake of the app is required for downstream data in the reporting engine to be useful.

The evaluation of the app’s feasibility, acceptability, and usability shall be conducted using the following scales and theoretical models and frameworks:

- Public Health England guidance on evaluating digital health products [30]
- Bandura’s model of health promotion by social cognitive theory will be used in the measurement of factors impacting the BCW framework development and validity, with specific emphasis on self-efficacy, perceived benefits, and perceived barriers [31].
- The reach effectiveness adoption implementation maintenance framework [32] to include information regarding the target population reach, potential for solution impact, adoption by target users, implementation consistency, and costs made during delivery and maintenance of the intervention
- Long-term adoption and suitability to further trials will be evaluated using the NASSS framework [26].

A total of 6000 (primary app users older than 65 years) will be recruited for evaluation. However, because recruitment will be done via the app store and advertised publicly, it is expected that the number of primary app users will exceed this number. We will randomly select 10% of the primary app users and 10% of secondary app users (adult users aged 18-65 years; participants will be drawn from sets collected at 1, 2, and 3 months poststudy commencement) for further qualitative investigation. A central study objective is to reach demographic saturation (ethnicity, social-economic background, and education) for study participants. Qualitative feedback will be measured through an examination of factors associated with app use and uptake. Participants will use the app online via smartphones at any location for, on average, 15-45 minutes per day during the implementation period. The qualitative evaluation will make use of in-app surveys and interviews. Study participants will be asked to take part in two interviews via Skype or telephone lasting 40-60 minutes conducted by the principal investigator (EM) and trained research staff. Interview questions will be asked within the context of how the participants interpreted the impact of the app. Interviews will be used to evaluate evidence-based strategies for engagement and BCTs, including self-monitoring, goal setting, physical activity and healthy eating support, personalized feedback and motivational strategies (eg, rewards, prompts, or gamification), and social support.

Skype and telephone conferences have been selected as methods of interviewing because participants are distributed regionally and adhering to social distancing regulations, and this is the most accessible means of interviewing participants. We will sample a sufficiently significant number of participants for qualitative interviews to provide sufficient insight into app impact. All those who opt for the study will undergo data analysis to avoid attrition bias. After being given information on the structure of the study to ensure that they understand it, participants will be asked to provide informed consent. Should participants opt not to have interview sessions audio recorded, detailed notes of those sessions will be taken and shared with participants at the end of the interview summarizing discussions. The full study design is under development and will be submitted for review in May 2020.

## Results

This paper summarizes the real time development of ADAPT-CAFÉ to share design principles that could be reused or extended by other app developers and scientists. In this section, we summarize the in-progress work plan for the solution.

### Work Plan

#### Phase 1: Discovery Phase

To ensure the build of a fit-for-purpose app, a project initiation document detailing user needs, the current state of the literature, and requirements specific to self-isolation and government policies surrounding the lockdown will be produced and agreed upon by all parties.

User stories and epics surrounding the accessibility requirements of older users and their families will be further developed to enable users with little to no digital skills to use the technology efficiently. Initial wireframes will be produced, and small focus group meetings will be held via Skype or Zoom to validate the defined problem and proposed solution.

This phase’s work has already commenced due to the urgent nature of the problem.

#### Phase 2: Alpha Phase Prototype

Focusing on the self-isolation challenge epic, a prototype participant interaction system and chatbot to aid social connection and physical and mental fitness for older people will be developed. The type and format of the data to be collected will be explored to ensure data interoperability with external data systems.

Concurrently, research ethical approval [33] will be sought at this stage from the University of Oxford to ensure that the solution can be implemented and evaluated in an ethical manner complying with privacy, data security, and other considerations.

#### Phase 3: Beta Phase Testing

The prototype app will be released to a small group of app testers. Qualitative feedback will be measured using the evaluation protocol designed in the alpha phase through Skype interviews of participants (families or peer groups and associated older stakeholders). Iterations to the app will be made based on suggestions by users. Iterative data analytics and fine-tuning of the data collection process will be conducted at this phase and aggregated with other data sources outside of the app to allow aggregate data analysis via the GE Healthcare Command Center. This work will continue throughout the development life cycle of the app to allow for continuous improvement of the app.

The technology development will follow a modular approach following the representational state transfer application program interface structure. Backend app databases will be hosted on the Amazon Web Services (AWS) infrastructure, complying with General Data Protection Regulation-compliant levels of data security and enabling big data processing (including voice and video transfer, storage, and analytics) as well as the deployment of machine learning modules.

At the core of the system, implementation is an artificial intelligence (AI)-powered chatbot agent, deployed with an open-source framework such as Amazon Lex, a service that allows the creation of intelligent conversational chatbots and is part of the AWS ecosystem. The integrated machine learning modules will give users a personalized experience to improve the quality of question prediction and response.

To cater to the needs of older adult users, a voice-enabled conversational AI will support accessibility and improve the ease of interaction. The app will use a voice-user interface that interacts with voice servers such as Amazon Alexa and delivers voice recognition, conversational dialogues, entity resolution, and memory.

At the second stage of the process after 1 year, the developed ecosystem will create a basis to further improve the technology for delivering advanced medical and lifestyle assistance for older and isolated people. The core data processing and storage engine are planned to be expanded with electronic health care records (EHR) data exchange gateway to connect with third-party EHR data providers and deliver personalized plans and recommendations, as well as automate schedules and programs for care and interactions. At that stage, the distributed ledger technology component is planned to further augment the work of independent communities via securing data exchange mechanisms and enabling token-based motivational schemes.

#### Phase 4: Live Phase

The app will go live on the Apple App store and Google Play store after beta iterations. The app will be marketed through social media channels and paid digital advertising. Continuous support and software updates will be made based on user feedback.

An evaluation study will be conducted using a combination of online surveys and qualitative video call interviews at various time points. A short-term time point will be examined for users after 5 days of app use to understand whether the app is successful in meeting the needs of users and improve and update the app to meet short-term user needs. A longer-term time point will be examined 2 months after the start of live app use to understand the attrition rate and long-term effects of using the app in the pandemic context.

**Figure 3:**
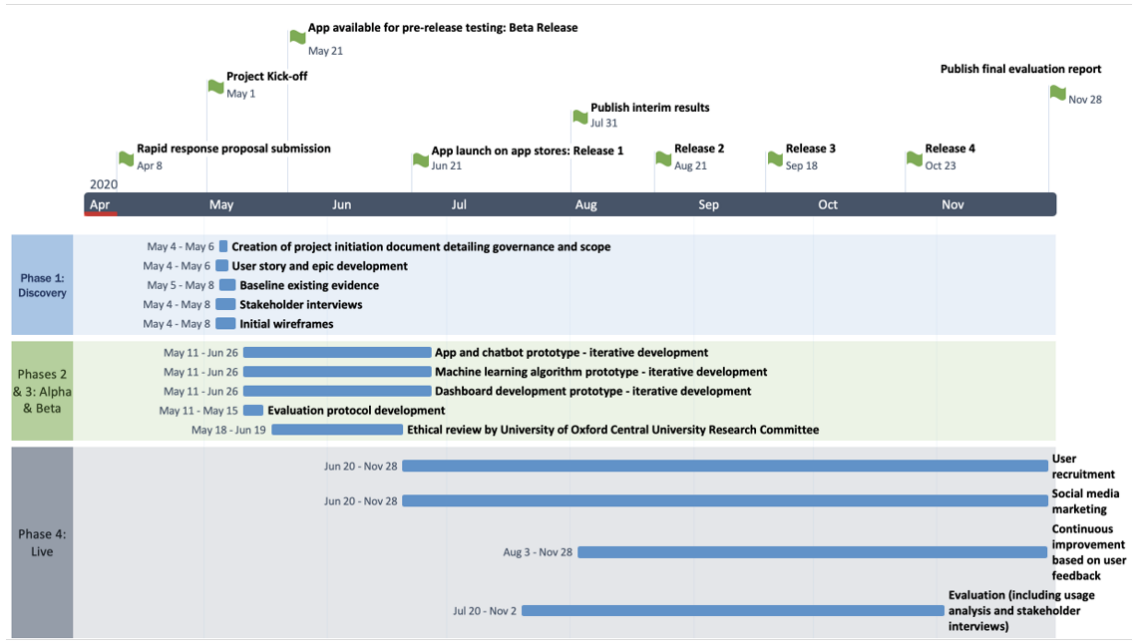
Proposed implementation timeline.

The results will be published in a peer-reviewed journal to share the experience in the development and feedback of the app and contribute to the broader community of researchers tackling the challenges arising from this worldwide pandemic. Figure 3 shows the proposed implementation timeline.

## Discussion

### Principal Findings

Mobile digital technology enables the capability to rapidly design, build, and deploy solutions with the capacity of connecting vast geographies of individuals. Despite the constraints of social distancing, such digital technology creates a capability for interconnectedness. The critical challenge, however, in the design of digital innovations is to construct them in a way that allows for evaluation and will assure the potential for long-term uptake and use. These issues are even more pressing with the context of this pandemic because resources must be deployed in a way that ensures a promise of effectiveness.

### Lessons Learned

This project was the genesis of brainstorming to a rapid-response call, which was developed hours before a submission deadline and subsequent iteration to four other rapid-response requests over 3 weeks. This pandemic has required the development of solutions in real time to respond to a public health emergency of international concern. It is challenging for academic institutions and funders to react responsively, particularly in medicine, because systems are designed to follow structures that ensure safety and evidence-based practice, which by their very design are methodical and time intensive. Despite these institutional barriers, however, the international clinical, academic, and industrial community have responded with speed during this crisis. The key lessons learned will be long-term enablement of what has worked for agile solution delivery and how we can embed these practices in care delivery in the future.

It is also worth considering other ideas that were not funded or unable to be developed due to constraints. A lesson learned for the community is how to channel these efforts and not merely rely on a meritocratic belief that the best solutions always present themselves. In a digital age, we can mobilize people, ideas, and resources in exponential ways, but making use of effort and its deployment is by no means simple.

### Strengths and Limitations of the Study

Our app development adopts user-centered design and takes into account evidence-based theories for the implementation of technology interventions in health and care. The composite of consideration for iterative development and system-wide thinking combined with a framework for evaluation during a rapid app development process is a strength of our approach. A limitation of our approach is that, due to the evolving nature of the current problem, it is unfeasible to follow a traditional scientific investigation format where the design would be finalized up-front and research ethics developed and approved before any software development was started. However, analysis of the efficacy and validity of our methods are the reasons we have submitted our work for international peer review while obtaining research funding for our solution.

### Further Research

The success and failure of digital solutions used during this pandemic will make valuable contributions to the literature. Lessons learned can be applied to influence future software engineering management of digital health solutions. Whether the authors can achieve the uptake and data sources intended will provide information about the best ways of combining real world and clinical data to inform potential case demand.

### Conclusions

This case study outlined a digital health agile requirements engineering approach to tackle a new and urgent issue arising from government measures to combat the COVID-19 worldwide pandemic. The proposed solution is to use a peer-to-peer engagement system and voice AI chatbot to connect older people with their family and friends and promote mental, physical, and social well-being. The testing and evaluation of the app will be reported in future studies.

## Abbreviations

ADAPT-CAFÉ: Activating Digital to Support Social Distancing COVID-19 Aware Family Engagement
AI: artificial intelligence
AWS: Amazon Web Services
BCT: behavior change techniques
BCW: behavior change wheel
COVID-19: coronavirus disease
EHR: electronic health care records
NASSS: nonadoption, abandonment, scale-up, spread, and sustainability
